# Video-enhanced informed consent improves patient comprehension in reverse total shoulder arthroplasty: a randomized controlled trial

**DOI:** 10.1016/j.jseint.2026.101735

**Published:** 2026-05-15

**Authors:** Paul D. Scheidegger, Franziska C.S. Altorfer, Bettina Hochreiter, Karl Wieser, Mazda Farshad, Samy Bouaicha

**Affiliations:** Department of Orthopaedic Surgery, University of Zürich, Balgrist University Hospital, Zürich, Switzerland

**Keywords:** Reverse total shoulder arthroplasty, Informed consent, Patient education, Educational video, Patient comprehension, Multimedia education, Orthopedic surgery

## Abstract

**Background:**

Informed consent for reverse total shoulder arthroplasty (rTSA) requires thorough patient understanding, yet only 29% of patients adequately comprehend pre-operative information. This study evaluated whether pre-operative educational videos enhance patient comprehension compared to standard verbal explanations alone and assessed impact on consultation time and demographic variations.

**Methods:**

This prospective randomized controlled trial included 60 patients undergoing rTSA, assigned 1:1 to video education or control groups. The video group viewed a 2-minute explanatory video before informed consent discussion; the control group received only verbal discussion. Afterward, all participants completed a 7-question test on procedural knowledge, benefits, and risks.

**Results:**

The video education group achieved significantly higher scores (5.70/7) compared to controls (4.67/7) (*P* = .002). Significant improvements were observed across all subgroups. Vulnerable subgroups, including older (*P* = .02), lower-educated (*P* = .007) participants, and non-native speakers (*P* = .04), also demonstrated benefit.

**Conclusion:**

An explanatory video before informed consent discussion significantly improved procedural comprehension compared to standard discussion alone. Vulnerable subgroups, older participants, non-native speakers, and those with lower educational attainment showed particular benefit. These findings support integrating video education into standard pre-operative rTSA protocols.

Reverse total shoulder arthroplasty (rTSA) has emerged as a well-established and effective treatment option for patients suffering from glenohumeral osteoarthritis or irreparable rotator cuff tears.^7^^,^^13^ Notably, the incidence of rTSA in the US escalated to over 60′000 surgeries in 2017, with numbers expected to increase further.^1^ More recent analyses indicate that the proportion of rTSA among all primary shoulder arthroplasties rose from 55% in 2016 to nearly 70% in 2020. Between 2011 and 2017, rTSA increased by 191.3%, and this exponential growth is projected to continue during the next decade.^14^^,^^20^

Pre-operative patient education and informed consent has been established as standard of care all over surgery, aiming to promote patient compliance, enhance satisfaction, and avoid lawsuits.^3^^,^^9^,^10^^,^^15^ However, many patients retain only a limited amount of the information conveyed during preoperative consultations with only 29% of the patients showing adequate comprehension of the provided information, whereas only 36% showed baseline knowledge regarding surgical risks.^3^ Furthermore, it is noteworthy that many patients are unaware of their deficits in comprehension and tend to overestimate their knowledge of their surgical procedure.^2^ Furthermore, the understanding of surgical consent can be affected by individual patient factors such as language barriers, low education levels, diverse ethnicity, or age potentially resulting in reduced patient comprehension.^6^^,^^12^ One relevant predictor of patient comprehension has shown to be the amount of consent time.^6^ However, extending the consent time in everyday practice is often challenging due to fully booked consultation hours.

To improve patients' understanding physicians started to use multimedia, such as educative videos (EVs), within the consent discussion to improve comprehension and satisfaction levels.^4^,^5^^,^^11^^,^^12^^,^^16^^,^^17^ For example, patients scheduled for lower extremity surgery, such as ankle surgery or knee arthroscopy, were shown a pre-operative EV resulting in a significant improvement in comprehension of their upcoming surgery.^16^^,^^17^ Similar findings were seen in patients undergoing arthroscopic shoulder surgery.^11^ In contrast, when looking at the understanding for total shoulder arthroplasty the use of pre-operative EV increased patient satisfaction but comprehension was not increased.^5^ However, no studies to date have investigated the impact of a pre-operative EV about rTSA, despite their increased use and challenging anatomical concept.

Therefore, the purpose of this prospective randomized study is to evaluate whether patients' comprehension can be enhanced by presenting an EV before the consent consultation for rTSA compared to purely verbal education. In addition, the study aims to assess potential variations in results based on patients' mother tongue, age, sex, and educational background. We hypothesized that the use of a pre-operative educational video would significantly improve patient comprehension of rTSA compared to verbal informed consent only, with particular benefit in vulnerable subgroups.

## Methods

### Study subjects

For this prospective randomized controlled trial, patients scheduled for rTSA at a single institution were recruited between April 2023 and January 2025. Recruitment was conducted in the hospital prior to pre-operative examination. All participants provided written consent prior to enrollment in the study. This study was approved by the Cantonal Ethics Committee of Zurich. The study was conducted in accordance with the principles of the Declaration of Helsinki.

### Inclusion and exclusion criteria

Inclusion criteria were patients who were scheduled for primary rTSA, a minimum age of 18 years, and a basic comprehension and speaking of the local native language, German, as the questionnaire was administered in native language. Patients with previous arthroplasty (anatomical or reversed) on the ipsilateral or contralateral shoulder joint were excluded. Other previous shoulder surgeries or arthroplasties of other joints were not considered exclusion criteria.

### Study design

The participants were randomly grouped into 2 groups. To the first group, a pre-operative EV on rTSA was shown in the patients' native language with subtitles prior to regular in person surgical consent discussion (EV group). To the control group (non-EV group), no EV was shown and a regular in person surgical consent discussion was done. All consent discussions, regardless of group allocation, were conducted by residents in accordance with a standardized institutional informed consent protocol.

**Figure 1:**
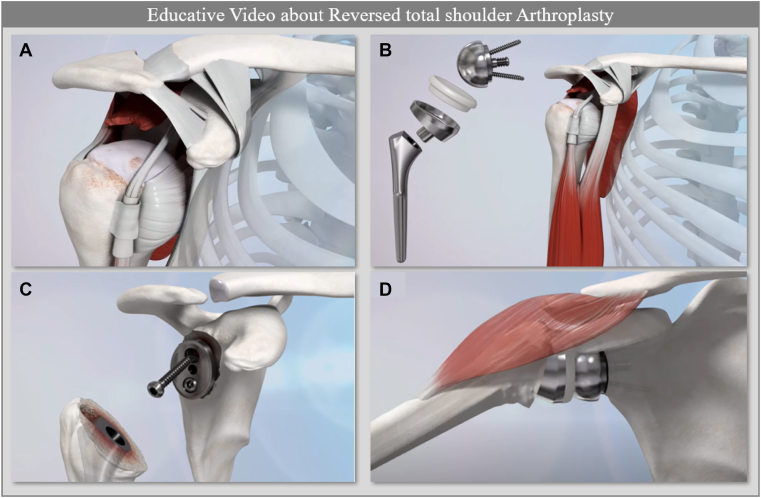
Representative snippets of the EV shown before surgical consent from the animated educational video illustrating relevant sections are represented in image (**A**), showing the surgical indication being for example a chronic rotator cuff tear; (**B**) representing the 3 components of the rTSA, including shaft, inlay, and head; (**C**) showing implantation of the glenoid component as well as the prepared humeral shaft; and (**D**) final implant positioning. *rTSA*, reverse total shoulder arthroplasty; *EV*, educative video.

The EV included audio and visual information about the technical procedure of rTSA and took 2:06 minutes. Representative scenes of the EV are shown in Figure 1. The EV can be accessed online (https://youtu.be/lRd013iFcN0). While watching the EV, participants were supervised to assure it was displayed correctly. However, no additional explanation was given during watching. After the informed consent discussion, participants from both groups completed the same questionnaire assessing their knowledge of clinical and technical aspects of rTSA. After finishing the informed consent discussion, each participant completed the questionnaire individually on a tablet, under supervision in case of any technical challenges.

### Comprehension questionnaire

The questionnaire was structured into 2 parts: (I) demographics and (II) comprehension assessment. (I) Demographics included age, sex, mother tongue, as well as level of education being lower education (compulsory education) or advanced education (apprenticeship or university). (II) The objective assessment included 7 yes/no items, with 5 questions developed de novo and 2 questions adapted from previously published literature on rTSA.^5^ The full questionnaire can be found in Table I.

**Table I:** Representative questions of the questionnaire, structured in 2 parts: (I) demographics, including age, sex, mother tongue, level of education, as well as questions regarding prior video viewing and previous shoulder surgery; and (II) objective assessment, containing 7 questions with the corresponding answers either being F or T.

No.	Question	Answer
Part I: Demographics		
1	How old are you?	
2	What is your sex?	
3	What is your native language?	
4	Was the consultation conducted in a language you understand?	
5	What is your highest level of education completed?	
6	Have you already watched a video about your surgery at home before today's consultation?	
7	Have you previously had surgery on your shoulder?	
Part II: Comprehension assessment		
1	In reverse total shoulder arthroplasty, the native joint anatomy is preserved.	**F**
2	In reverse total shoulder arthroplasty, a humeral stem is implanted into the humerus.	**T**
3	The goal of reverse total shoulder arthroplasty is to improve pain and joint function.	**T**
4	Complications arise only from poorly performed surgery.	**F**
5	The prosthesis functions just as well as a healthy shoulder joint.	**F**
6	I will have to wear a sling for three weeks after the surgery.	**F**
7	I will continue to see improvements in my shoulder function for up to two years after the surgery.	**T**


*F*, false; *T*, true.

### Randomization

The randomization to the 2 groups was done based on the date patients were scheduled for surgery, meaning that the participants undergoing surgery between April and August 2023 were shown the EV (EV group), while the second group between October 2023 and January 2025 received a regular informed consent without additional EV (non-EV group). Due to logistical reasons, this randomization was considered the most feasible and reliable. The surgeons conducting the informed consent discussion were not informed about group affiliation of the patients.

### Methods of measurement

The 7 statements about rTSA from the (II) objective part of the questionnaire were rated by the patient as either true or false. Participants received 1 point per correct answer. Primary outcome measure was patients' comprehension score, defined as number of correct answers in the questionnaire, ranging from possible 0-7 points. Therefore, a maximum score of 7 and a minimum score of 0 was possible.

### Data analysis

Data were analyzed using Excel. For group comparisons of knowledge scores f-tests were calculated, followed by independent *t*-tests assuming equal or unequal variances based on the f-test results. For effect size quantification, Cohen d values were calculated. Statistical significance was set at *P* < .05.

## Results

A total of 60 patients were enrolled in this study, with 30 assigned to each group (EV and non-EV). The overall mean score on the postconsent questionnaire was 5.18 ± 1.42 (95% confidence interval [CI]: 4.82, 5.54). The EV group achieved a significantly higher mean score (5.70 ± 0.88, 95% CI: 5.39, 6.01) compared to the non-EV group (4.67 ± 1.67, 95% CI: 4.07, 5.26; *P* = .002, Cohen d = 0.78) as shown in Table II.

**Table II:** Overall and group specific postconsent comprehension scores.

Variable	Overall	Non-EV	EV	*P* value
Comprehension Score	5.18 ± 1.42	4.67 ± 1.67	5.70 ± 0.88	.002

*EV*, educative video.

Values are presented as mean ± standard deviation for all participants (overall), non-EV and EV group.

## Demographics

The mean age was 72 ± 10 years overall, with no significant difference between the EV group (73 ± 8 years) and the non-EV group (72 ± 12 years; *P* = ns). Gender distribution was significantly different between groups: the EV group consisted of 63.3% male participants (n = 19) and 36.7% female participants (n = 11), while the non-EV group had 30.0% male (n = 9) and 70.0% female participants (n = 21; *P* = .0097).

The distribution of native vs. non-native speakers did not differ significantly between the EV group (86.7% native, n = 26; 13.3% non-native, n = 4) and the non-EV group (70.0% native, n = 21; 30.0% non-native, n = 9; *P* = .117).

Educational level was comparable between groups. In the EV group, 26.7% (n = 8) had lower education and 73.3 (n = 22) had advanced education, compared to 20.0% (n = 6) and 80.0% (n = 24), respectively, in the non-EV group (*P* = .542). No participants reported having received no formal education. Demographic information for both groups are shown in Table III.

**Table III:** Demographic characteristics of participants in the EV and non-EV groups.

Characteristics	Overall (n = 60)	EV (n = 30)	Non-EV (n = 30)
Age (yr) mean ± SD	72 ± 10	73 ± 8	72 ± 12
Sex, (n)			
Male	46.7% (28)	63.3% (19)	30.0% (9)
Female	53.3% (32)	36.7% (11)	70.0% (21)
Native language, (n)			
Native speaker	78.3% (47)	86.7% (26)	70.0% (21)
Non-native speaker	21.7% (13)	13.3% (4)	30.0% (9)
Education level, (n)			
Lower education	23.3% (14)	26.7% (8)	20.0% (6)
Higher education	76.7% (46)	73.3% (22)	80.0% (24)

*EV*, educative video.

Values are presented as mean ± standard deviation (SD) for continuous variables and as counts with corresponding percentages for categorical variables.

### Comprehension score

Comprehension scores ranged from 2 to 7 across all participants, with no participant scoring 0 or 1. Twelve patients (20.0%) achieved the maximum score of 7. The EV group demonstrated a narrower score distribution (4 to 7) compared to the non-EV group (2 to 7).

In the EV group, the majority of participants (63.3%, n = 19) scored either 6 (46.7%, n = 14) or 7 (16.7%, n = 5). In addition, 26.7% (n = 8) scored 5 and 10.0% (n = 3) scored 4. No participants in the EV group scored below 4.

In the non-EV group, 33.3% (n = 10) of participants achieved comprehension scores of 6 or higher, with 10.0% (n = 3) scoring 6 and 23.3% (n = 7) scoring 7. Lower scores were more prevalent: 13.3% (n = 4) scored 5, 23.3% (n = 7) scored 4, 23.3% (n = 7) scored 3, and 6.7% (n = 2) scored 2. Notably, 30.0% (n = 9) of non-EV group participants scored below 4.

### Comprehension score subgroup analysis by age

Within each age group, mean scores did not differ significantly between participants ≤75 years and those >75 years of age. In the EV group, participants ≤75 years (n = 17, mean age = 67 ± 5.7 years) achieved a mean score of 5.88 ± 0.78 (95% CI: 5.51, 6.25), while those >75 years (n = 13, mean age = 81 ± 4.02 years) scored 5.46 ± 0.97 (95% CI: 4.94, 5.99; *P* = .09). In the non-EV group, participants ≤75 years (n = 17, mean age = 64 ± 9.54 years) scored 4.88 ± 1.76 (95% CI: 4.04, 5.72), while those >75 years (n = 13, mean age = 82 ± 6.36 years) scored 4.38 ± 1.56 (95% CI: 3.54, 5.23; *P* = .21).

When comparing between EV and non-EV groups, both age categories showed significantly higher scores in the EV group. Participants ≤75 years scored higher in the EV vs. non-EV group (*P* = .02, Cohen d = 0.73), as did participants >75 years (*P* = .02, Cohen d = 0.83). The effect size was larger in the older age group (Table IV).

**Table IV:** Postconsent comprehension scores stratified by age, language, and education.

Variable	Age (Yr)		Language		Education	
	≤75	>75	Native	Non-native	Lower	Higher
Comprehension Score						
Non-EV	4.88 ± 1.76	4.38 ± 1.56	4.83 ± 1.70	4.14 ± 1.57	3.67 ± 1.63	4.83 ± 1.71
EV	5.88 ± 0.78	5.46 ± 0.97	5.73 ± 0.92	5.50 ± 0.58	5.63 ± 0.92	5.73 ± 0.88
P value	.02	.02	.01	.04	.007	.015

EV, educative video.

Values are presented as mean ± standard deviation for the non-EV and EV group.

### Comprehension score subgroup analysis by language

Native speakers and non-native speakers achieved comparable mean comprehension scores. In the EV group, native speakers scored 5.73 ± 0.92 (95% CI: 5.38, 6.08) compared to 5.50 ± 0.58 (95% CI: 4.93, 6.07) for non-native speakers (*P* = .26). In the non-EV group, native speakers scored 4.83 ± 1.70 (95% CI: 4.13, 5.52) compared to 4.14 ± 1.57 (95% CI: 2.98, 5.31) for non-native speakers (*P* = .34).

Both native speakers (*P* = .01, Cohen d = 0.68) and non-native speakers (*P* = .04, Cohen d = 1.02) scored significantly higher in the EV group compared to the non-EV group (Table IV). The effect size was larger among non-native speakers.

### Comprehension score subgroup analysis by education level

Within each intervention group, scores did not differ significantly between participants with lower vs. higher education levels. In the EV group, participants with lower education scored 5.63 ± 0.92 (95% CI: 4.99, 6.26) compared to 5.73 ± 0.88 (95% CI: 5.36, 6.10) for those with higher education (*P* = .39). In the non-EV group, participants with lower education scored 3.67 ± 1.63 (95% CI: 2.36, 4.97) compared to 4.83 ± 1.71 (95% CI: 4.15, 5.52) for those with higher education (*P* = .07).

Both educational levels demonstrated significantly higher scores in the EV group. Participants with lower education scored higher in the EV vs. non-EV group (*P* = .007, Cohen d = 1.55), as did participants with higher education (*P* = .015, Cohen d = 0.65). The effect size was notably larger among participants with lower education (Table IV).

## Discussion

This study showed that the use of an EV in patient consent for rTSA results in a significantly higher patient comprehension compared to the control group, with a moderate to large effect size (Cohen d = 0.78, *P* = .002). The EV group achieved a significantly higher average comprehension score with a narrower score range, while the average was lower in the control group with a wider score range.

The here presented results aligned with previous video education studies in orthopedic surgery. Hoppe et al^11^ studied patient's comprehension of arthroscopic procedures of the shoulder after watching an educational video and reported a relevant higher rate of correct responses (87% vs. 56%) in the video vs. the non-video group. Similar results were found for the understanding of knee arthroscopy using a video-only approach (without physician discussion) and found 78.5% vs. 65.4% correct responses.^17^ The here presented study of rTSA combining EV plus physician discussion achieved 81.4% vs. 66.7% (Cohen d = 0.78). Despite differences in procedures, assessment methods, video content, duration, and educational approaches, all studies demonstrated consistent substantial benefits of video-based education for patient comprehension of orthopedic surgeries.

However, it was noteworthy that Fasulo et al^5^ found no significant differences in knowledge retention between video and control groups for total shoulder arthroplasty at both immediate (87.1% vs. 87.2%, Cohen d = –0.01) and 6-week follow-up (86.7% vs. 87.7%, Cohen d = –0.09) assessments. Nevertheless, they reported higher patient satisfaction with the video education.^5^

Using video technology was beneficial for patients of different age groups in terms of knowledge acquisition and retention. Despite potential concerns about technology acceptance in older participants, the EV was beneficial across all age groups, with both younger and older participants achieving significantly higher comprehension scores. Not only was there an increased comprehension in all age groups, but also the EV effect was particularly strong in older patients. These results imply that older demographic subgroups can benefit greatly from video-based consent consultation.

Regarding patients' language proficiency, both native and non-native speakers in the EV group demonstrated significantly higher comprehension scores. Notably, non-native speakers exhibited a markedly larger effect size, suggesting they benefited especially from the video-based instruction. This increased effectiveness potentially reflects the capacity of visual information to compensate for language barriers. In previous research, the presence of subtitles have provided additional comprehension support for non-native speakers, showing that video captions are beneficial to all viewers, especially to non-native speakers.^8^ These findings have relevant implications for patient care in multicultural health care environments, where effective communication strategies are essential to ensure comprehension and informed decision-making across diverse linguistic backgrounds.

Another potential factor influencing patients' comprehension of medical content is their level of education, with higher education being associated with better understanding of informed consent.^18^ In the present study, participants across both educational levels benefited from the video-based approach. However, those with lower educational attainment showed even greater improvement when using an EV compared to their more highly educated counterparts, indicating enhanced benefit from EV. This suggests that videos can bridge educational gaps, which is important because studies have shown that patients with lower levels of education understand consent forms less than those with higher levels of education.^19^ From ethical and justice perspectives, this is crucial, as all patients should have equal access to comprehensible health information and receive informed consent tailored to their individual needs and capabilities.

Limitations of this study include methodological constraints, temporal limitations of the knowledge assessment, content restriction of the EV, and generalizability concerns. Firstly, although the study was designed with blinding in mind, complete blinding was not achieved, as surgeons may have learned of participants' group allocation during consent consultation. Furthermore, the Hawthorne effect may have influenced the results, as participants' awareness of being observed and assessed may have led to increased attention and engagement during the consent consultation, which could have artificially elevated comprehension scores. In addition, a residual novelty effect in the EV group, arising from the additional stimulus of the educational video, cannot be fully excluded and may have further contributed to the observed between-group difference. Secondly, knowledge assessment was conducted only once, immediately following the consent consultation, raising questions about whether results would have been comparable in a later assessment. Thirdly, the EV was relatively brief (2:06 minutes), covering only the basics regarding indication and surgical procedure for rTSA. Furthermore, consent discussion was not conducted by the responsible surgeon, but by various residents. However, oral consent was done based on a standardized institutional protocol ensuring equivalent informational coverage across all participants. Although all participants reported not having watched any video regarding their surgery, prior consultation of other educational materials remains a possible source of bias. In addition, the date-based group allocation does not constitute true concealed randomization and represents a recognized methodological limitation, as it can be manipulated, potentially leading to selection bias. This was a deliberate pragmatic decision driven by logistical constraints within this single-center clinical setting. Importantly, the 2 groups did not differ significantly in age, language, or education level. Although a significant difference in sex distribution was observed, with 63.3% men and 36.7% women in the EV group and 30.0% men and 70.0% women in the non-EV group, there is no evidence that sex has a significant influence on comprehension scores, suggesting that the allocation did not introduce relevant systematic bias. Furthermore, no formal a priori power analysis was performed. The sample size of 30 patients per group was informed by comparable studies examining educational interventions in orthopedic surgery.^5^^,^^11^ A post hoc sensitivity analysis using GPower (version 3.1) confirmed that, given n = 30 per group and α = 0.05 (two-tailed), the minimum detectable effect size at 80% power was Cohen d = 0.74, and the observed effect of d = 0.78 corresponds to an achieved power of 84%. We acknowledge that the absence of a formal a priori power analysis represents a methodological limitation of the present study. Finally, as this was a single-institution setting, the generalizability of the results remains uncertain.

Future research could focus on implementation and practicability, such as cost-benefit analyses or time-efficiency studies. Furthermore, future studies should incorporate a pretest/post-test design to formally account for baseline differences in procedural knowledge. In addition, patient-specific factors such as long-term retention or individual preferences could be investigated. Content optimization and standardization in terms of length, structure, and content represent another potential field for research. The transferability of this approach to other medical fields warrants further investigation.

## Conclusion

This study demonstrated that using an EV before the informed consent discussion significantly improved patients' comprehension of rTSA compared to standard discussion alone. Vulnerable subgroups, including older participants, non-native speakers, and those with lower educational attainment, showed particular benefit from the video-based instruction. These findings support the integration of video education into standard pre-operative protocols for rTSA in clinical practice.

## Declaration of generative AI and AI-assisted technologies in the manuscript preparation

During the preparation of this work, the authors used Claude (Anthropic) in order to search for literature and edit text. After using this tool/service, the authors reviewed and edited the content as needed and take full responsibility for the content of the published article.

## Disclaimers:

Funding: No funding was disclosed by the authors.

Conflicts of interest: The authors, their immediate families, and any research foundations with which they are affiliated have not received any financial payments or other benefits from any commercial entity related to the subject of this article.
